# Addressing Climate Change Communication: Effective Engagement of Populations for Climate Action in the US and Globally

**DOI:** 10.5334/aogh.2900

**Published:** 2020-05-22

**Authors:** Ana Viamonte Ros, Regina LaRocque, Rachel Fortinsky, Patrice Nicholas

**Affiliations:** 1 Florida International University, US; 2 Massachusetts General Hospital, US; 3 Johns Hopkins University, US; 4 MGH Institute of Health Professions, US

## Abstract

**Background:**

Communication about climate change is critical in addressing the greatest public health challenge of our time. Public health professionals must convey the human implications of climate change and educating populations regarding climate change as a threat to the health and wellbeing of people globally. Effective communication to engage individuals, communities, and populations is critical to debate as we focus on the most urgent public health problem of our time.

**Objective:**

Public health professionals are aware of the deleterious health consequences related to climate change; however, key segments of the population are not. This paper addressed key concepts related to climate change communication.

**Methods:**

Databases were searched including PubMed, CINAHL, and Scopus from 2015 to 2020 to obtain the most recent relevant literature using search terms that included climate change, climate communication, climate action, and climate change engagement.

**Findings:**

Climate change communication as viewed through the lens of Six Americas—a national survey that categorized people regarding their beliefs about climate change from those who are Dismissive, Doubtful, Disengaged, Cautious, Concerned, or Alarmed is a valid perspective for engaging populations in climate communication and climate action.

**Conclusions:**

Using the framework developed by the George Mason University Center for Climate Change Communication and the Yale Program on Climate Change Communication, we suggest that adopting this framework from a US perspective to a global perspective and surveying across countries and context is imperative to advance global understanding of the impact of climate change on health.

## Background

Climate change represents an urgent public health problem which is growing in scope globally. Despite recent political challenges related to addressing climate change, the public health community is galvanized to engage in safeguarding the health of the world’s people and alleviating the suffering related to the deleterious climate-related health consequences that are emerging [1]. One of the most important climate action challenges is engaging in communication within our health professions communities and with our patients, families, communities, and populations. All of the health professions—medicine, nursing, and others—are poised to serve as experts in addressing the intersection of climate and health. From air quality issues related to greenhouse gas emissions (GGEs), vectorborne diseases, heat stress in vulnerable populations and occupational workers, and mental health consequences that result from disasters, health professionals must lead in communication efforts to highlight the urgent challenges that we face.

As noted by the Yale Program on Climate Change Communication [2], the most essential and leading strategy to communicate effectively is to *know thy audience*. In other words, in order to transmit a resonating message, climate change advocates must recognize their listener’s unique background—psychological, cultural, and political—which likely influences their eagerness, or even willingness, to reduce GGEs and adopt a lifestyle that improves planetary health and health co-benefits. *Knowing thy audience* is critical in engaging health professionals in understanding the intersection of climate and health, as well as galvanizing provider engagement in education with patients, families, communities, and populations.

In an effort to improve effective climate change communication, *Global Warming’s Six Americas* was developed to understand the unique views of the American public regarding climate change [2]. This research identified six categories, each with a distinct attitude toward climate change. First launched in 2008, the Six Americas identified six audiences from a large nationally representative survey of American adults, which included survey measures of the public’s beliefs, attitudes, risk perceptions, motivations, values, policy understanding, behaviors, and barriers to action. The Six Americas distinguishes specific attributes which result in differing levels of engagement related to their understanding of climate change as an overarching issue and its intersection with health.

In a 2015 survey [3], most Americans reported an understanding that global warming poses a health danger. However, for most Americans, their understanding did not expand much deeper. In fact, few understood the types of harm climate change inflicts and the groups who are most vulnerable to its effects. This may explain the moderate support the survey respondents indicated regarding an expanded public health response. This finding suggests that primary care physicians, nurses and other health professionals, and public health officials have key roles in educating the public about the health relevance of climate change.

## Findings

**Figure 1:**
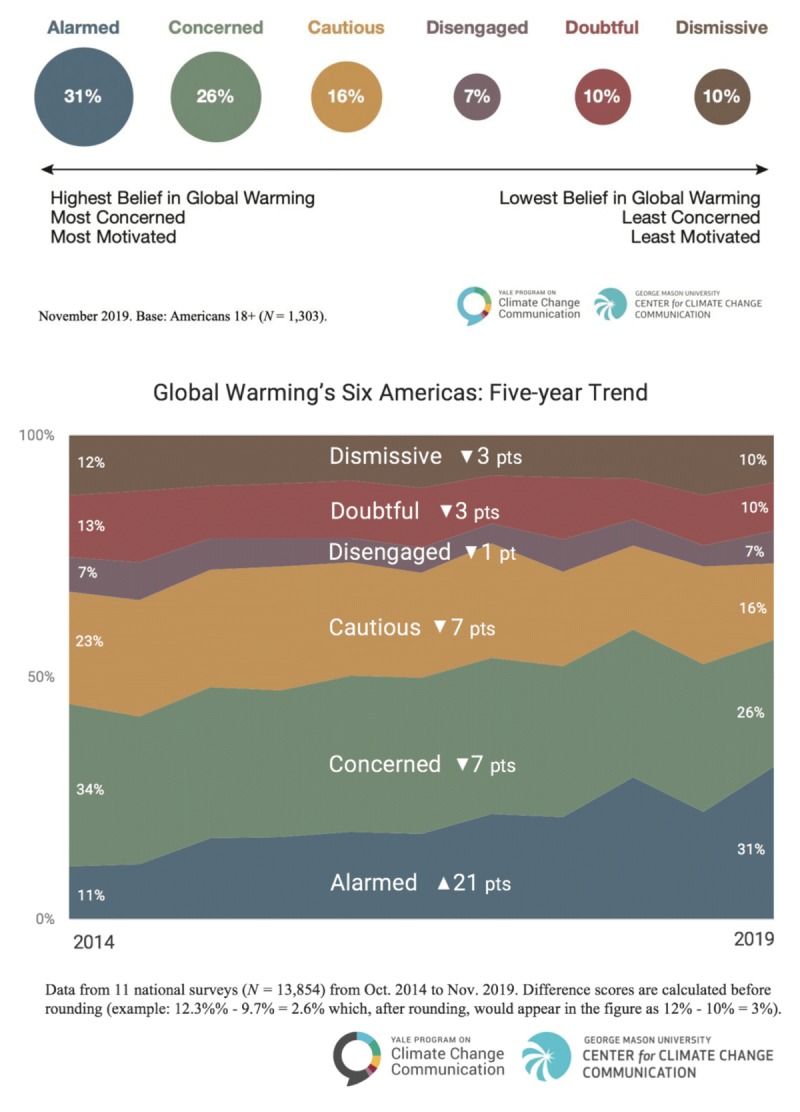
November 2019 Six Americas Survey Results and Global Warming’s Six Americas Five-Year Trend.

Findings from the Yale Communication Project in partnership with the George Mason University Center for Climate Change Communication [2,4] suggest that the American public underestimates how many *other* Americans think global warming^1^ is happening; thus they underestimate the social consensus on global warming—a fact that yields opportunities for health care providers to engage in climate and health discussions. Further, they note that Americans on average estimate that only 54% of other Americans think that global warming is happening; however, the surveys suggest that 69% of Americans do. Regarding the categories of survey respondents, in November 2019, the *Alarmed* (31%) are fully convinced of the complexity and severity of climate change and are already addressing individual, community, and advocacy efforts [2,4]. The *Concerned* (26%)—the category that represents the largest of the six Americas—are also among those who are convinced about global warming, but are not fully engaged in climate action. Three other Americas—the *Cautious* (16%), the *Disengaged* (7%) and the *Doubtful* (10%)—are differing categories, and none are actively involved in climate action due to their lack of understanding or belief in global warming. The final America—the *Dismissive* (10%)—do not believe that global warming is happening and oppose any governmental or public health efforts to reduce GGEs. It is interesting to note that in November 2019, a shift in those who indicated they were in the *Alarmed* category increased to 31% from 26% supporting the growing concern about climate change in the US and the shift in five-year trends (Figure 1).

A recent national survey suggested that the American public underestimates how many *other* Americans think global warming^1^ is happening [4]. In two 2019 reports, the authors note that Americans underestimate the broadening social consensus on global warming—estimating that only 54% of other Americans think global warming is happening, when in fact 69% of Americans do. Further they note that only about one in six Americans (16%) think global warming is not happening and that Americans who think global warming is happening far exceed those who think global warming is not happening by more than a 4 to 1 ratio [5]. In the most recent report published in January 2020 by the Yale Program on Climate Change Communication and George Mason University Center for Climate Change Communication, survey findings indicated that for the first time, the *Alarmed* category increased from 11% in 2014 to 31% in 2019 [6].

This represents a critical opportunity for health professionals to engage with patients, families, communities, and populations who may have a greater readiness to understand the importance of mitigation, adaptation, and resilience efforts amidst our increasing climate health challenges.

## Conclusion

Building on the importance of education of health professionals, it is critical to integrate climate content in curricula in health professions programs in medicine, nursing, pharmacy, physician assistant studies, physical therapy, occupational therapy, nutrition, and other programs. These health education initiatives have been identified as essential to prepare for current and future health challenges and to bring climate discussions into provider-patient encounters [7]. Recently the American Medical Association adopted a policy statement in support of education about climate change for all physicians and medical students, a movement that continues to garner support nationwide in medicine, nursing, and other health professions curricula [8,9]—a movement that has also been galvanized by the American Public Health Association. Finally, nearly all health professions are gaining understanding of the complex, chronic, and deleterious consequences on health in our climate-changing world, thus communication among US and global populations is important to engage the public in the critical dialogue on climate and health.

## Footnotes

The term that resonates more frequently for the public is global warming; thus this term was used in the national survey.
